# Mental Health Peer Worker Perspectives on Resources Developed from Lived Experience Research Findings: A Delphi Study

**DOI:** 10.3390/ijerph19073881

**Published:** 2022-03-24

**Authors:** Shannon Li, Anne Honey, Francesca Coniglio, Peter Schaecken

**Affiliations:** 1 School of Health Sciences, University of Sydney, Sydney, NSW 2006, Australia; shannonli98@hotmail.com; 2 Private Practitioner, Sydney, NSW 2000, Australia; francescad.coniglio@gmail.com; 3 New Horizons, Sydney, NSW 2113, Australia; pschaecken@newhorizons.org.au

**Keywords:** lived experience research, co-produced research, mental health, peer work, health resources, Delphi technique, consensus

## Abstract

Lived experience research is potentially useful for assisting the recovery journeys of people experiencing mental health challenges, when presented in user-friendly formats. Consumer peer workers are ideally placed to introduce such resources to the people they work with. This study sought to explore the perspectives of expert consumer peer workers on the potential use of lived experience research resources in peer work practice. In particular: (1) what research topics would be most useful; and (2) what considerations are important for developing user-friendly and useful resources using findings from this research. A hybrid Delphi study was conducted. Eighteen expert peer workers participated in online group interviews, which included a semi-structured discussion and modified nominal group technique. These were followed by two rounds of surveys, which focused on prioritising the identified topics. Participants identified 47 topics suitable for lived experience research resources, 42 of which reached consensus as useful for consumers. A priority list of topics for use in peer work was identified through examination and grouping of peer worker rankings of the usefulness of resources for their work with consumers. The highest priority topics were as follows: developing and maintaining social networks; how peer workers can support consumers in their recovery journey; having choice with medications and participating in the decision-making process; and knowing your rights and responsibilities. Participants noted, however, that the usefulness of each topic ultimately depended on individual consumer’s needs. They highlighted that a variety of formats and presentation were required to reach diverse consumer groups.

## 1. Introduction

Lived experience research in the mental health field is research conducted by, and with, people with direct experiences of mental health challenges [1]. As such, it is likely to be highly relevant, holistic, and trustworthy [2,3,4]. It also aligns with the values of the consumer movement: “nothing about us, without us” [5].

While lived experience research is critical for positive system change [6], it also has the potential to be directly useful for consumers in their recovery journeys. For example, lived experience research often brings together the stories and experiences of consumers around a specific experience. Active engagement with and learning from others’ experiences can instill hope [7], which is fundamental to recovery [8]. This potential, however, is predominantly untapped. Many consumers are not even aware that lived experience research exists [9]. Additionally, research findings are often inaccessible due to academic jargon and lack of open access [10,11].

A recent study [12] sought to address this by developing a selection of lived experience research articles into six accessible and engaging resources for consumers, such as a podcast and magazine. These resources were introduced to 38 consumers at a public mental health service and evaluated using mixed methods. The study found that between 80 and 91% of participants who used each resource reported that it had improved their perceptions towards their future and their recovery journey. Furthermore, between 85 and 100% of participants said they would recommend each resource to others. In qualitative feedback, participants reported that interacting with the resources had made them feel more hopeful, empowered, and connected with others.

In the study, people were introduced to the resource by peer workers, who were seen as the ideal people to disseminate lived experience research findings [13]. Peer workers are mental health workers who draw upon their lived experience of mental health challenges and recovery to support consumers in their recovery [14]. They adopt an empowerment and recovery-oriented approach in their practice, which involves supporting the person holistically to live a meaningful life, despite symptoms [15,16,17]. This is in contrast to the traditional biomedical model, which focuses on diagnoses and treating symptoms [17]. Peer workers are well positioned to disseminate lived experience research findings, as they work with consumers on an equal level and practice from a lived experience base [14]. This means they are likely to have a clear understanding of consumers’ needs and aspirations, can tailor their approach to best engage the person, and understand what can support them with aspects of their recovery. Peer workers who used the lived experience research resources found them relevant to their daily practice [13].

Overall, previous research has indicated that lived experience research resources had great potential for use by peer workers with consumers. However, people’s preferences for specific resources varied depending on their needs, interests and circumstances [12], suggesting that the number and variety of resources should be expanded to cater for a wider variety of needs and interests. To date, no literature exists to guide the development of lived experience research resources, including topics, formats and presentation strategies. Such guidance is needed to ensure that resources developed from lived experience research findings will be used in everyday practice by peer workers, and are experienced as helpful by consumers. Thus, the current research sought to identify the topics likely to be most used by consumer peer workers in their daily practice, and explore considerations around resource development.

This study sought to answer the following research questions:

1. What lived experience research topics do expert consumer peer workers identify as useful for their work with consumers in their practice?
2. What considerations are important when developing resources from lived experience research findings?

Answering these questions will facilitate the future development of lived experience resources to support both the peer work role and consumer recovery.

## 2. Materials and Methods

### 2.1. Study Design

This study was conducted as part of the STELLER project (Supporting the Translation into Everyday Life of Lived Experience Research), which aims to promote lived experience research by translating the findings into user-friendly resources for consumers’ daily lives. The research team for this study comprises researchers with and without lived experience, with Author 4 also having extensive peer work experience. The study was approved by the University of Sydney Human Research Ethics Committee (project number 2020/820).

A hybrid Delphi method [18] with three stages of data collection and analysis was used for this study. The Delphi method is an iterative process that utilises the knowledge and experiences of key experts to address issues that have limited research or agreement, as well as identify priorities for action [19,20,21,22]. While the traditional Delphi method [23] involves multiple rounds of surveys, the hybrid Delphi [18] replaces the first round of surveys with group interviews including a semi-structured discussion segment and a modified nominal group technique (NGT). 

The hybrid Delphi [18] addresses drawbacks of traditional Delphi methods: the considerable amount of time needed between questionnaires which increases risk of participant dropout [19,20], and the inability to engage in discussion to clarify issues. The latter was particularly relevant in this emerging and innovative field of research, where some explanation and clarification of concepts were needed. Previous studies conducted using the hybrid Delphi method demonstrated high participant retention rates and number of ideas generated compared with traditional Delphi methods [18]. 

### 2.2. Sampling and Recruitment

Given the suitability of peer workers in disseminating lived experience research findings and the relevance to their practice, 18 consumer peer workers were recruited as key experts for this study. An expert is defined here as someone who is acknowledged by their peers as being experienced, skilled and knowledgeable in their field.

The inclusion criteria for participation in this study were as follows:

1. Having at least one year’s experience as a consumer peer worker;
2. Recommended as experts in the field of peer work by a colleague;
3. Currently working in adult mental health services.

The authors contacted peer work managers, senior peer workers, the state peer workforce committee and peer work researchers. These people were identified from researchers’ networks and publicly available information. These contacts were requested to identify expert peer workers and provide them with an invitation to participate. Interested peer workers contacted Author 1 or submitted an online Expression of Interest. Participants were also asked to invite other peer workers they considered experts. These strategies resulted in recruitment of 18 expert peer workers. While recommendations differ, 10 to 30 participants are generally recommended for a Delphi study [24,25], with 10 to 15 participants for a homogeneous sample of participants [22]. Participants were allocated to group interviews based on their availability, and all participants provided written informed consent.

### 2.3. Data Collection and Analysis

As Delphi is an iterative process, data collection and analysis for each stage will be described sequentially.

#### 2.3.1. Stage 1: Group Interviews

Data collection for Stage 1 was undertaken via group interviews that combined a semi-structured group discussion with a modified NGT [18]. The NGT component primarily addressed question 1 by identifying useful lived experience research topics, while the semi-structured discussion component primarily addressed question 2, exploring how lived experience research findings could be translated into accessible resources to maximise usefulness and acceptability.

Group interviews were conducted online via Zoom to adhere to COVID-19 safety restrictions and for participants’ convenience. Each group interview was planned to have around four participants, to allow sufficient time for each person’s ideas to be fully expressed. While five to ten participants per group is seen as desirable by Landeta et al. [18], this recommendation was based on the assumption that interviews would be conducted face-to-face. Recommendations in health research for numbers of participants in focus groups conducted via videoconferencing are lacking, but market research sources indicate that three to six participants is more appropriate, due to challenges with sustaining people’s attention and participation in an online environment [26,27].

Group interviews were facilitated by Authors 1 and 2. The researchers began by recapping the purpose of the research, defining lived experience research, and showing examples of resources developed from the findings of lived experience research that had been used in the previous study. Participants were invited to consider and informally discuss the potential benefits, challenges and considerations for using lived experience research in their work with consumers.

Next, a modified NGT was used to gather ideas and priorities for the development of resources from the findings of lived experience research. Participants were given time to independently write their thoughts about topics that would be useful in the daily lives of consumers they worked with. Each participant then shared their responses, as Author 1 took notes. The resulting list of suggested topics was shared and discussed, and corrections or additions were made.

When all groups had been conducted, the lists of lived experience research topics from each group were compared. Similar ideas were merged, resulting in the identification of 47 discrete topics. 

The interviews were transcribed and analysed using constant comparative analysis [28] to identify broader views and considerations around using lived experience research. This involved examining the data line by line and assigning one or more concepts or codes to each piece of data. Subsequent data were compared to previously coded data; if the concept was the same, the data were added to that code; if not, a new code was made. Throughout this process, codes were refined to capture the main themes of the data. Codes were then compared to other codes to examine underlying themes or ideas; similar codes were unified to make higher level, broader codes. This comparison of codes allowed the researchers to understand the relationships between codes. Authors 1 and 2 coded the first two group interviews and compared codes to improve theoretical sensitivity and reach consensus. NVivo 12 [29] was used to manage the coding.

#### 2.3.2. Stage 2: Delphi Survey 1

Participants were emailed a survey, which asked them to rate the 47 topics identified in Stage 1 on how useful they would be as lived experience research resources for consumers to use in their daily lives (1 = not useful, 2 = a little useful, 3 = useful, 4 = most useful, with ‘unsure’ as an opt out option). Participants were also asked to comment on their answers and to rate how likely (not likely, somewhat likely, likely, very likely) they were to use resources about the topics they saw as ‘useful’ or ‘most useful’ with consumers in their peer work practice. Participants were also provided with a summary of the considerations for resource development identified in Stage 1, and invited to offer further comments or feedback.

The average usefulness score for each topic was calculated. Consensus on the usefulness of a topic was set at 3.00 (useful) or above (e.g., [30]). Specific comments made about each topic and comments explaining why participants were most or least likely to use a resource were summarised.

#### 2.3.3. Stage 3: Delphi Survey 2

Participants were presented with a summary of the results from the first survey, including average usefulness scores and comments about each topic, general comments on usefulness, and a graph detailing the spread of scores for each topic. 

As only 5 out of 47 topics from Survey 1 failed to reach consensus, and these were closely clustered with the other items, all items were included in the second survey. The aim became the prioritisation of topics that would be most useful to peer workers in their practice. This acknowledged peer workers’ strong position in disseminating the research to consumers. Participants were asked to select the top 10 lived experience research topics they believed would be most useful as resources for peer workers when working with consumers. In informing their prioritisation, participants were asked to consider the range of settings in which peer workers work, as well as the current availability of other resources on the topics. Participants were asked to comment on the choice of their top five selections. Because comments revealed that two items (developing and maintaining social networks; and link between social connectedness and mental health) were being seen the same way, these items were combined.

Following data collection, two measures were calculated for each topic to ascertain consensus on the highest priorities for development of lived experience research resources: (1) the total number of participants who selected the topic in their top ten; and (2) weighted totals (first choice = 10 points, second = 9 points, and so on). These were examined to ascertain clusters of priority. 

## 3. Results

Of the 18 peer workers recruited, 15 people participated in all parts of the study. One person participated in the group interview only, one person completed both surveys but was unable to attend the group interview, and one person participated in the group interview and first survey but did not complete the second survey. Information collected from the Expressions of Interest indicated that most participants (*n* = 17) worked in New South Wales (NSW), and had current or previous experience in a range of practice settings such as public (*n* = 18) and private (*n* = 1) sectors, non-government/community-managed organisations (*n* = 13), rural services (*n* = 1), inpatient services (*n* = 9), and community services (*n* = 12). Years of experience as a peer worker ranged from one to over 10 years.

Due to scheduling difficulties, numbers in the group interviews varied from expected, with two participants being interviewed alone, as the other participants in their groups were unable to attend. Seven interviews were held in total (see Table 1 for the distribution of participants). 

Participants reported many benefits to using findings from lived experience research in their work with consumers. They thought that it had the potential to bring hope; provide validation for people’s recovery; create a sense of belonging; uncover multiple perspectives on different issues; and provide opportunities for consumers to hear other people’s success stories. It was seen as relevant, relatable, and potentially inspiring, with the credibility or status of research holding value among consumers. As one participant explained:

*“I think [lived experience research] would be really helpful, because I’m just one person, and that’s just my own story. And it’s so different from everyone else’s.”*

*(P13)*

Participants indicated that lived experience research was consistent with peer work principles like mutuality, reciprocity and empowerment. They believed that resources created from the findings from this research could act as a springboard for connection and interaction, and would be a good addition to peer workers’ tools for practice because of the lived experience focus:

*“There are a lot of resources if you have time to search for them on the internet, but the resources might not be necessarily tailored to peer support workers … and a lot of things can be quite clinical.”*

*(P17)*

### 3.1. Lived Experience Research Topics

In the NGT, participants identified 47 lived experience research topics, the findings from which they thought would be useful in the daily lives of consumers. In Survey 1, 42 of these topics were rated as ‘useful’ or ‘very useful’ by more than 75% of participants, with an average score above 3 (‘useful’), and a highest score of 3.65 out of 4. Most participants reported that they were likely (23.5%) or very likely (64.7%) to use resources about the topics rated as ‘useful’ or ‘most useful’ with clients in their peer work practice. 

Survey 2 indicated four groupings of topics prioritised by usefulness in peer work practice. The four highest priority topics were selected by over 50% of participants in their top 10, and had a weighted total above 50. The next five high priority topics were selected in the top 10 by at least 33% of participants, and had a weighted total above 20. Topics rated in the top 10 by between two and four participants (*n* = 27) were deemed medium priority, and those rated in the top 10 by one or no participants were deemed low priority (*n* = 10). The topics, grouped according to priority, along with the number of participants who selected each topic in their top 10 and the weighted total, are listed in Table 2. The topics that failed to reach consensus in Survey 1 are also marked in Table 2. Examples of participants’ comments explaining their prioritisation of the highest and high priority topics are provided in Table 3.

Participant comments indicated that some lower rated topics, such as help with hoarding and looking after yourself following bereavement, while seen as very useful for some consumers, were viewed as less commonly useful. Other topics, while likely to be useful to consumers, were seen by some participants as better addressed by other health workers such as social workers. These topics were as follows: accessing financial support; managing finances; getting into work or study; managing and balancing work or study; and getting housing and accommodation.

### 3.2. Considerations for the Development of Lived Experience Research Resources

Participants discussed many considerations for developing lived experience research resources. Peer workers advised that resources needed to be developed according to principles of accessibility; choice and variety; usefulness across settings; empowerment and recovery orientation; interaction and connection; and cultural applicability.

#### 3.2.1. Accessibility

Participants believed it was important for the resources to be accessible to a broad audience. Resources should avoid jargon, and accommodate people’s differing literacy levels and cognition. However, participants emphasised being careful not to over-simplify, which may be infantilising or demeaning.

*“I wouldn’t even talk to a child the way some [other] material does, let alone an adult … I think it’s actually quite disrespectful … So when I say simplify, I don’t mean dumb it down.”*

*(P11)*

Length of time needed to use a resource should also be considered, as some consumers may find it repetitive, or have a shorter attention span.

*“You want to be thorough, but also concise … [People] already do a lot of questionnaires [and] feedback things … It depends on the person, but most people [in our service] are not too keen to sit down for any great length of time.”*

*(P14)*

On the other hand, more detail about the research should be made available for people who express interest in it to facilitate their engagement:

*“Some people may get at a level … who want to actually read the whole journal article. So, providing multiple layers of the same research at different literacies.”*

*(P1)*

When designing digital resources like websites and apps, they should be easy to use, with an intuitive and visually appealing interface. Having the resources all in one place would be convenient for both peer workers and consumers to access.

*“From a digital perspective, just making sure … [to consider] the user experience, and have easy-to-navigate webpages, mobile-optimised aspects … [be] search engine optimised, and [have] easy-to-share options.”*

*(P4)*

Resources should also be accessible for people with disabilities, such as hearing and vision impairments. The cost of producing or using the resources should be as minimal as possible.

#### 3.2.2. Choice and Variety

Participants highlighted the importance of having choice and variety of resources, to cater for different people’s learning styles, interests, and where they are in their recovery. Allowing consumers to choose which resource works for them also facilitates autonomy. Thus, it would be useful to have resources on the same topic available in different formats, for example, podcasts, workbooks, videos, and artistic formats.

*“I don’t think there will ever be this one resource that will be the right resource for everyone. Lots of people are auditory learners, visual learners, kinetic, so there’s never gonna be just this one. The choice, that’s what makes it.”*

*(P7)*

Preferences may differ depending on demographics such as age and gender, for example, younger generations may prefer digital formats. Novelty is also important:

*“There’s a lot of information that is on a poster, or written … and sometimes that doesn’t really mean a lot to people. Like, if you give them so many posters and flyers and brochures, in time, they just become like junk mail.”*

*(P17)*

#### 3.2.3. Usefulness across Settings

Relatedly, participants felt that there was a need to design resources that can be used across settings, including inpatient settings. They emphasised that some consumers in these settings would engage well with the resources, if given the opportunity.

*“There are a lot of consumers [in inpatient settings] who are actually complaining about not getting enough information, or being bored and having nothing to do … A lot more places are starting to have kiosks and mobile phone use on wards, which would help people to actually make use, in their downtime, of these resources.” (P5)*

#### 3.2.4. Empowerment and Recovery Orientation

In addition to giving people choice, resources should be empowering for consumers to use, for example, by using strengths-based and empowering language; being holistic; acknowledging the recovery process; and being genuine in their portrayal of people.

*“The language used throughout [the resources] needs to be recovery-oriented, and created by people with lived experience … Very often, even if something has good intent and meaning, if the language isn’t appropriate, that has the effect of turning people away. Whatever is produced … [needs] to focus maybe less on certain diagnoses and certain treatment options, but really tap into being trauma-informed.”*

*(P8)*

Essentially, resources should look at the person holistically, for example, exploring what the person wants in the future, and not be overly focused on clinical aspects.

*“Sometimes, when we put the focus on the mental health that consumers are experiencing, it can kind of lock them in. So, instead of focusing on whatever’s happening in the moment … it’s looking towards where they want to go. So, in a way, looking towards where you want to go helps with what you’re experiencing in the moment.”*

*(P9)*

It is also important that resources acknowledge that recovery or improvement is a process, and should avoid being ‘preachy’, especially when addressing habits that may not be healthy.

*“There’s a lot of pressure on things like, “You need to stop smoking”, “You need to eat well” … I think that just comes as a by-product of recovery. When people learn to take care of themselves, then that kind of just comes into play … It will just happen, as opposed to the pressure of focusing, “I’m quitting smoking right now”.”*

*(P13)*

Lastly, when representing people in the resources, they should be portrayed in diverse situations with genuine emotions, so that people can relate to them. For example, pictures showing everyone smiling can seem unrealistic.

*“Not everybody is going to be sparkling every day … it just depends on where that person is on their recovery journey. They may actually relate better to that [neutral] picture rather than someone who’s smiling.”*

*(P16)*

#### 3.2.5. Interaction and Connection

Participants expressed the need for the resources to be designed in a way that encourages interaction and connection with others. For example, considering formats that are more ‘hands-on’, to help initiate conversations.

*“Having some sort of joint resource that a person can do with somebody … just opens up a lot of avenues for conversations that might not be easy to have when you first meet.”*

*(P17)*

Resources that could be used in group settings were also frequently discussed by participants. Additionally, an element of fun was seen as helpful in building connections. These factors would help create a more relaxing or informal atmosphere.

*“The one thing that is missing out of people’s lives is the element of fun, which is why I go to games. Because some people won’t say no to a game.” *

*(P16)*

#### 3.2.6. Cultural Applicability

Finally, participants thought that resources should not only be available in different languages, but culturally relevant and appropriate, especially since perceptions around mental health can vary across cultures.

*“When you’re looking at CALD people or Indigenous peoples, the language around how you present that information would definitely need to be considered.” *

*(P4)*

Adopting a co-design approach by partnering with people with specific and relevant lived experience to develop resources was thought to help ensure that resources are culturally relevant. A couple of participants mentioned that research from the context in which it would be used (in this case, Australia) would be particularly useful in some cases, as experiences of recovery are directly impacted by the contexts within which people live and access care and support.

## 4. Discussion

The findings (a) support the relevance of lived experience research to peer work practice and its development; (b) suggest an agenda for lived experience research and resource development; and (c) imply a need for additional funding and support for lived experience research. 

Participants indicated that the use of lived experience research fits in well with peer work practice. Many principles, such as mutuality, reciprocity, authenticity, empathy and empowerment, are shared [14,31], indicating a natural alignment and greater likelihood of using the resources in peer work practice. Both peer work and lived experience research focus on harnessing people’s lived experience to offer support and learning to others. Thus, peer workers can draw upon this research to offer different perspectives, with strong credibility and collective wisdom. Peer workers are well placed to disseminate this research to consumers, because of their ability to develop meaningful relationships with consumers [32]. They are able to tailor their approach to individual preferences, and can support consumers to feel at ease and open to trying the resource in the first place [13]. The high and highest priority topics that peer workers saw as most useful for their practice with consumers had common themes of social connection; empowerment; self-advocacy; and promoting and understanding recovery for different groups of people. These themes are aligned with recovery-oriented practice, enabling peer workers to better support consumers in their recovery journeys, even when they are often in biomedical-based settings [15].

The benefits participants described of using lived experience research in their practice support the opinions of the peer workers who were involved in disseminating the test resources to consumers [13]. This indicates a wider understanding among peer workers of the potential benefits of using lived experience research. It is acknowledged, however, that peer workers are not the only people who could deliver resources on lived experience research findings. Some participants saw other health professionals as better equipped to discuss certain topics with consumers, such as finance and housing. This presents a potential scope for lived experience research resources that are delivered by different health professionals that best fits their area of expertise. Regardless of who presents the resources, however, their use will be either facilitated or inhibited by clinicians and health managers who make decisions about funding and resource allocation. Peer workers have reported feeling overloaded and lacking resources for practice [33]. By developing and enacting policies that provide peer workers with the time, funding and autonomy to use innovative recovery-based resources, managers will nurture the role of peer workers within the mental health system. 

The findings demonstrate considerable scope for lived experience research to investigate a variety of topics. Nearly all topics were seen as useful to consumers, including those that were quite specific, such as hoarding or bereavement. This was reflected in the results of Delphi survey 1, where 42 out of 47 topics reached consensus. Furthermore, while the highest priority items were a clear grouping based both on number of appearances in the top 10 and weighted totals, the distinction between the lower groups was less clear. Some of the items selected by fewer people had a higher weighted total than those selected by more people, indicating that those who did select these items, rated them highly. This emphasises the scope for resources on a wide variety of topics relevant to peer work practice. After all, as participants stated, the usefulness of a resource depends on the individual’s needs, as people’s circumstances, interests, and where they are in their recovery journey will vary. 

It is currently unclear, however, whether, and to what degree, the topics identified in this study have been addressed in lived experience research to date. Current lived experience research is primarily systems-focused, highlighting the delivery and quality of services, combatting stigma [1,6]; guidelines on involving consumers in research [11,34]; mental health training in education [35]; capacity building, and changes to legislation [10,36]. This research offers important strategic and systemic insights, contributing to organisational change. However, research designed to be used directly by consumers in their daily lives is less common. Further, the research that does exist is difficult to systematically identify. Lived experience research is difficult to search and distinguish due to varying definitions of lived experience research and levels of consumer involvement [37], combined with a lack of consistent keywords used for publications identifying lived experience research and researchers. Stigma, negative attitudes and beliefs towards mental health challenges, fear of discrimination, negative impacts on career prospects, and potential professional licensing issues, can lead to consumer researchers not disclosing their lived experience [10,34,38]. Thus, the involvement of consumer researchers sometimes goes unstated in articles, and when it is stated, it can be unclear how lived experience has informed the research. A proximal follow-on from this study is the identification of existing lived experience research around the identified topics. This identification will require not only systematic and complex database searching, but appealing to and communicating with networks of lived experience researchers throughout the world. Lived experience researchers who have engaged in research on the topics identified in this study are encouraged to consider the potential for translation of their research into resources for consumers and to enlighten the authors about their work. Given the observed scarcity of lived experience research on topics relevant to consumers’ daily lives, additional lived experience research on these topics is also likely to be warranted. 

The research team is currently working toward creating a suite of lived experience research resources, including modules for peer worker training. The findings not only provide guidance on topics for our team and others to inform the development of peer worker-delivered resources, they will also inform how those resources are produced. Considering accessibility, choice and variety, usefulness across settings, empowerment and recovery orientation, interaction and connection, and cultural applicability, will ensure that the resources are appropriate for peer work practice. 

In order to tap into its full potential in supporting people’s recovery, broader support and recognition for a wide variety of lived experience research is needed. A range of structural and societal barriers to undertaking lived experience research currently exist. For example, there is a lack of funding, and lived experience researchers may have difficulty getting their research published and peer-reviewed [39], the latter affecting its perceived credibility among other researchers and clinicians. Other challenges include lack of support and training for consumer researchers, time pressures, inflexibility of policies and procedures [37,38]; and a lack of opportunities for mentoring and career progression as a consumer researcher [34,37]. Specific funding, training and support for lived experience research should be built into research structures, for example, by universities and health services. The use of lived experience research can also be increased through improved visibility, as many people are unaware that lived experience research exists [9] and, for the reasons discussed above, it can be difficult to find. Attention is needed to enable the reliable identification of lived experience research, for example through the use of standard key words. However, increased visibility will best be achieved when lived experience research enjoys the funding, support structures and status that it deserves.

### 4.1. Strengths and Limitations

A major strength of this study was having peer worker and lived experience expertise on the research team from inception. Another was the identification of priorities by people with lived experience themselves. Considerable previous research has identified differences between the priorities of service users and professionals around health care issues, for example, what outcomes are important [40] and patient safety priorities [41]. Differences in research priorities between consumers and health professionals or academics without lived experience have also been highlighted, e.g., [11,42,43]. For example, research has suggested that consumers place higher value than professionals on social and psychological compared to biomedical research [11]; applied compared to basic research [44], and research on services users’ perceptions of illness and treatment and the health and welfare of carers [45]. Lived experience perspectives are clearly integral to developing an agenda for lived experience research resources.

The study’s inductive approach, with topics and considerations identified and prioritised by expert peer workers themselves, increases the likelihood that resources created on the identified topics will be used by peer workers, thus achieving dissemination to consumers. However, as the focus was on peer workers and their role in disseminating the research, Delphi survey 2 asked participants to select the most useful topics as resources for peer workers when working with consumers, rather than usefulness for consumers directly. The nuance between the two questions can be seen in the second highest priority topic “How peer workers can support consumers in their recovery journey”. While important, this topic may not necessarily be as high priority if rated by consumers (who are not peer workers). Future research with consumers is needed on a wider scale to find out what lived experience research topics they are interested in.

This study had a high retention rate; 16 out of 18 participants completed the final survey, improving rigour, especially as dropouts are common in Delphi studies [22]. However, scheduling issues, which were often last minute, meant that three of the interviews could actually be considered dyadic rather than group interviews. These share similar reliance on and process of interactions between participants as group interviews, collect similar data, and have some benefits in terms of detail and quantity of data collected from each person [46,47]. Further, two of the interviews had only a single person, as the other participants were unable to attend. This could have affected the ideas generated in those interviews, due to the lack of interaction with other participants. However, these issues are unlikely to have greatly affected the analysis as saturation was reached by the seventh interview. While the sample size of this study is standard for a Delphi study, it is still relatively small, and most participants were from NSW and lived in metropolitan areas. Findings may differ across states and countries.

### 4.2. Future Directions

In summary, this research is part of a program aimed ultimately at developing a substantial suite of resources based on lived experience research on topics that are both helpful within consumers’ daily lives and suitable for use with peer workers. The current research has identified topics likely to be useful in peer work practice and considerations for resource development. However, several further interim steps are needed to achieve our goal, including validating the usefulness of topics with consumers directly, identifying existing lived experience research, and enlarging the lived experience research base from which to draw.

## 5. Conclusions

This study identified the lived experience research topics that, according to peer workers, would be most useful as resources to use with consumers in their practice. These topics may be prioritized for development of resources as they are most likely to be used in peer work practice; however, lower rated topics should not be ignored, as individuals’ needs will vary. This study also described many important considerations when developing the resources, to ensure they are accessible and empowering to use, while facilitating connections with others. There is a clear need for funding of lived experience research that is directly relevant to consumers’ daily lives. Peer workers are well placed to effectively disseminate the research to consumers and can use lived experience research to develop their practice and unique identity.

## Figures and Tables

**Table 1:** List of participants in each group interview.

Group No.	Participants
1	Participants 1–4
2	Participants 5–6
3	Participants 7–11
4	Participants 12–13
5	Participants 14–15
6	Participant 16
7	Participant 17

**Table 2:** List of lived experience research topics by priority.

Topic	No. of Appearances in Top 10 (*n* = 16)	Weighted Total	Priority
Developing and maintaining social networks	11	91	
How peer workers can support consumers in their recovery journey	10	75	Highest
Having choice with medications and participating in the decision-making process	9	61	
Knowing your rights and responsibilities	9	51	
How recovery can look different for different groups of people, e.g., age, culture	6	29	
Looking after yourself when you don’t feel up to it	6	28	
Developing healthy boundaries	6	27	High
Strategies for self-advocacy	6	24	
Self-care and what it means to different people	6	23	
What is trauma	4	30	
Strategies for navigating the mental health system	4	27	Medium
Finding the right health professional, e.g., GP, psychologist, etc. *	4	27	
Experiences and benefits of different therapies, e.g., pet therapy, acceptance and commitment therapy, cognitive behavioural therapy, dialectical behavioural therapy, sensory modulation	4	24	
How to become a peer worker	4	22	
Thriving after trauma (post traumatic growth)	4	21	
Coping with grief and loss	4	21	
What promotes recovery	4	19	
Getting housing and accommodation	4	16	
Participating and contributing to improving the mental health system	4	16	
Using daily activities to promote mental health	4	15	
Giving feedback to services *	3	27	
Finding out about medications and their side effects	3	24	
What is recovery	3	17	
Relationship between physical health and mental health	3	16	
Getting the best out of a doctor or specialist visit	3	16	
Getting into work or study	3	14	
Developing assertiveness	3	12	
Strategies to promote a positive sense of self	3	6	
Managing symptoms for specific conditions, e.g., anxiety, depression	2	12	
Managing strong emotions	2	11	
Reaching out for help	2	10	
Transitioning from hospital to community and daily life	2	8	
Wellness plans	2	8	
Meditation and mindfulness	2	7	
Value and benefits of goal-setting	2	5	
Role of religion, faith and spirituality in mental health and recovery *	2	4	
Managing finances	1	9	Low
Help with hoarding	1	8	
Practical ways of healthy eating	1	8	
Accessing financial support	1	6	
Managing the side effects of medication	0	0	
Looking after yourself following bereavement	0	0	
Transitioning to life after being in gaol	0	0	
Using music to enhance recovery	0	0	
Managing and balancing work or study *	0	0	
Managing bullying, including cyberbullying *	0	0	

* = Topics that failed to reach consensus in Delphi survey 1.

**Table 3:** Participants’ top priority topics for peer work with example comments.

Topic	Example Comments
Highest Priority	
Developing and maintaining social networks	“Helping people develop social skills is paramount, as it is connectedness that best helps people recover. The more healthy relationships a person has, the better their mental health outcome.” (P11)
How peer workers can support consumers in their recovery journey	“[This] resource would be brilliant to help consumers better understand the ways a peer worker can be of assistance.” (P2)
Having choice with medications and participating in the decision-making process	“Gaining knowledge and power over the role that medication takes in a person’s life, and that it is their right to have control over what happens to their body, is so important.” (P5)
Knowing your rights and responsibilities	“As people often bring up their concerns around lack of rights and decision-making, it is a resource that I would introduce early in my interactions with people (unless they are too distressed or preoccupied) to have those conversations.” (P8)
High Priority	
How recovery can look different for different groups of people, e.g., age, culture	“[I would use this] every day, during group conversations.” (P8)
Looking after yourself when you don’t feel up to it	“I would use this resource regularly, because it is essentially about cultivating self-love in order to practice regular self-care/nurture. The skill of self-love often has to be learnt later in life, and is an essential part of recovery.” (P12)
Developing healthy boundaries	“Boundaries are so very important!” (P3)
Strategies for self-advocacy	“[I would use this] when someone is under the Mental Health Act, coming up to a tribunal, or an important meeting for their recovery.” (P13)
Self-care and what it means to different people	“Self-care is very important and an easy conversation starter, however not all people have the same self-care techniques, and it would be helpful to explore new ideas with people.” (P17)

## Data Availability

Not applicable.
